# Risk of prostate cancer after detection of isolated high-grade prostatic intraepithelial neoplasia (HGPIN) on extended core needle biopsy: a UK hospital experience

**DOI:** 10.1186/1471-2490-9-3

**Published:** 2009-05-27

**Authors:** Paras B Singh, Caroline M Nicholson, Narasimhan Ragavan, Rosemary A Blades, Francis L Martin, Shyam S Matanhelia

**Affiliations:** 1 Lancashire Teaching Hospitals NHS Foundation Trust, Fulwood, Preston, UK; 2 Centre for Biophotonics, Lancaster Environment Centre, Lancaster University, Bailrigg, Lancaster, UK

## Abstract

**Background:**

High-grade prostatic intraepithelial neoplasia (HGPIN) is a precursor lesion to prostate cancer (CaP). UK-based studies examining the occurrence of isolated HGPIN and subsequent risk of CaP are lacking. Our aim was to assess the occurrence of HGPIN in a regional UK population and to determine whether in a retrievable cohort of such patients that had repeat extended core biopsies, there was an elevated risk of CaP.

**Methods:**

A retrospective analysis of the pathology database was conducted at our institution (Lancashire Teaching Hospitals NHS Foundation Trust) for prostate biopsies recorded between January 2001 and December 2005 (all extended core biopsies). Those patients with isolated HGPIN on 1^st ^set of biopsies were identified and, their clinical characteristics and pathological findings from subsequent biopsies (if any) were determined. The risk of CaP on subsequent biopsies based on presenting baseline PSA was stratified.

**Results:**

Of 2,192 biopsied patients, there were 88 cases of isolated HGPIN of which 67 patients underwent one or more repeat biopsies. In this repeat-biopsy group, 28 CaP diagnoses were made. Age at first biopsy (*P *< 0.001), higher mean baseline prostate-specific antigen (PSA) (*P *< 0.005) and higher mean change in PSA (*P *< 0.05) were predictive of CaP detection on repeat biopsies. PSA ranges and their associated predictive values for cancer were: 0 to 5 ng/ml – 11%; 5 to 10 ng/ml – 34%; 10 to 20 ng/ml – 50%; and > 20 ng/ml – 87.5%.

**Conclusion:**

Based on our results, we recommend delaying the 1st repeat biopsy at low PSA range but to have a shorter interval to repeat biopsies at intermediate and higher PSA ranges.

## Background

High-grade prostatic intraepithelial neoplasia (HGPIN) is a precursor lesion to prostate cancer (CaP). Like CaP, the incidence of HGPIN increases with age and has a higher prevalence in African-American men [1]. HGPIN is more frequently found in prostates with cancer than those without [2] and, tends to be multifocal and located in the peripheral zone [3]. Similar genetic and molecular changes in HGPIN and CaP have been described [4]. Studies prior to the mid-1990s that examined cancer risk on subsequent prostate biopsies after diagnosis of isolated HGPIN showed a 27% to 100% elevated risk. Since the widespread adoption of extended core biopsy techniques, it has been suggested that there is a lower risk of CaP detection on repeat biopsies [5,6]. In fact, some studies now cast doubt on whether isolated HGPIN is associated with a high risk of CaP on repeat biopsies [5,7,8]. Reduced rates of CaP detection on repeat biopsies have been attributed to widespread prostate-specific antigen (PSA) screening resulting in early, small-volume cancer at diagnosis, and adoption of extended core biopsy techniques that sample more extensively the lateral regions of the prostate [5].

Almost all the recent studies that examine the risk of CaP detection following diagnosis of isolated HGPIN are based on US populations where PSA screening is widespread [6,9]. The corresponding UK-based experience examining the occurrence of isolated HGPIN and subsequent risk of CaP detection on repeat biopsies remains to be determined. The aim of this retrospective study was to exploit the pathology database in a UK hospital in order to evaluate the occurrence of isolated HGPIN in a specific regional population and, to determine whether in a retrievable cohort of such patients who had repeat biopsies, there was a subsequent elevated risk of CaP.

## Methods

With appropriate approval from the institutional audit department, a retrospective analysis of the pathology database was conducted at our institution (Lancashire Teaching Hospitals NHS Foundation Trust) for prostate biopsies recorded between January 2001 and December 2005. Those patients during this period with isolated HGPIN on 1^st ^set of prostate biopsies were identified. In these men with isolated HGPIN, the clinical characteristics and pathological findings from subsequent biopsies (if any) were also determined. Patient characteristics between those that went on to develop CaP and those that were found to have benign histology on subsequent biopsies were compared (unpaired Student *t*-test). All *P*-values given are two-tailed.

A further sub-group analysis looking at the subsequent risk of CaP based on the level of presenting PSA was performed to determine whether the risk was purely due to HGPIN or possibly due to concurrent CaP that was not biopsied at the time of 1^st ^biopsy. A Kaplan-Meier type estimator plot was generated using GraphPad Prism software (Version 4) based on this PSA sub-group data; diagnosis of CaP (on repeat biopsies) was assessed as the endpoint of interest.

## Results

During this period, extended core needle prostate biopsies were performed in a cohort of 2,087 men, of which 88 men (4.2%) had isolated HGPIN and 972 (46.6%) were diagnosed with CaP. In the 88 patients with isolated HGPIN on 1^st ^set of prostate biopsies, the median age was 68 y (range 53 y to 84 y). Their mean PSA before the 1^st ^set of prostate biopsies was 11.45 ng/ml (median 8.5; range 0.6–68.2) and they had a median of 12 core prostate biopsies (range 8 to 14).

Of the 88 patients with isolated HGPIN, 67 patients underwent one or more repeat biopsies at our institution. In those who had repeat biopsies, CaP was subsequently diagnosed in 28 patients (41.8% of 67 patients). Twenty-four (32.8%) were found to have CaP on 1^st ^set of repeat prostate biopsies at a median interval of 13.5 months. The number cores taken on the initial prostate biopsy and the number of cores with HGPIN on the 1^st ^biopsy were not associated with subsequent CaP detection (data not shown). The isolated HGPIN was originally reported as unifocal in 20 patients (71%) who were subsequently diagnosed with CaP on repeat biopsies.

Patient characteristics between those that went on to develop CaP and those that were found to have benign histology on subsequent repeat biopsies were compared (Table 1). Those who were subsequently diagnosed with CaP were significantly older (y; *P *< 0.001) and had a higher mean PSA (*P *< 0.005) at 1^st ^set of prostate biopsies. Patients that remained CaP-free had on average 1.4 repeat biopsies (range 1 to 3) at a median interval of 13.6 months (range 1 to 57 months) while, those that developed CaP had on average 1.25 (range 1 to 3) biopsies at a median interval of 14.5 months (range 1 to 42 months). There was a significantly higher change in mean PSA (*P *< 0.05) in those that were found to have CaP on repeat biopsies. Gleason 7 or higher tumours were detected in 35.7% of these patients who were subsequently diagnosed with CaP (Table 1).

**Table 1:** Comparison between patients with benign and malignant histology on repeat prostate biopsies

**Patient characteristics**	**Remained benign (n = 39)**	**Developed CaP (n = 28)**	***P*-value**
**HGPIN at 1^st ^set of biopsies**			
Mean age (y)	63.5	69.8	< 0.001
Mean PSA (ng/ml)	8.4	17.5	< 0.005
Mean number of biopsy cores	11	10.54	0.3
Unifocal HGPIN	27	20	
			
**At repeat biopsies**			
Mean number of repeat biopsies (range)	1.4 (1–3)	1.3 (1–3)	
Mean interval (months) to 1^st ^repeat biopsies	7.38	15.11	< 0.0005
Mean change in PSA (ng/ml)	0.5	4.8	< 0.05
			
**Gleason score**			
< 7	na	18	
7	na	7	
> 7	na	3	

**Figure 1:**
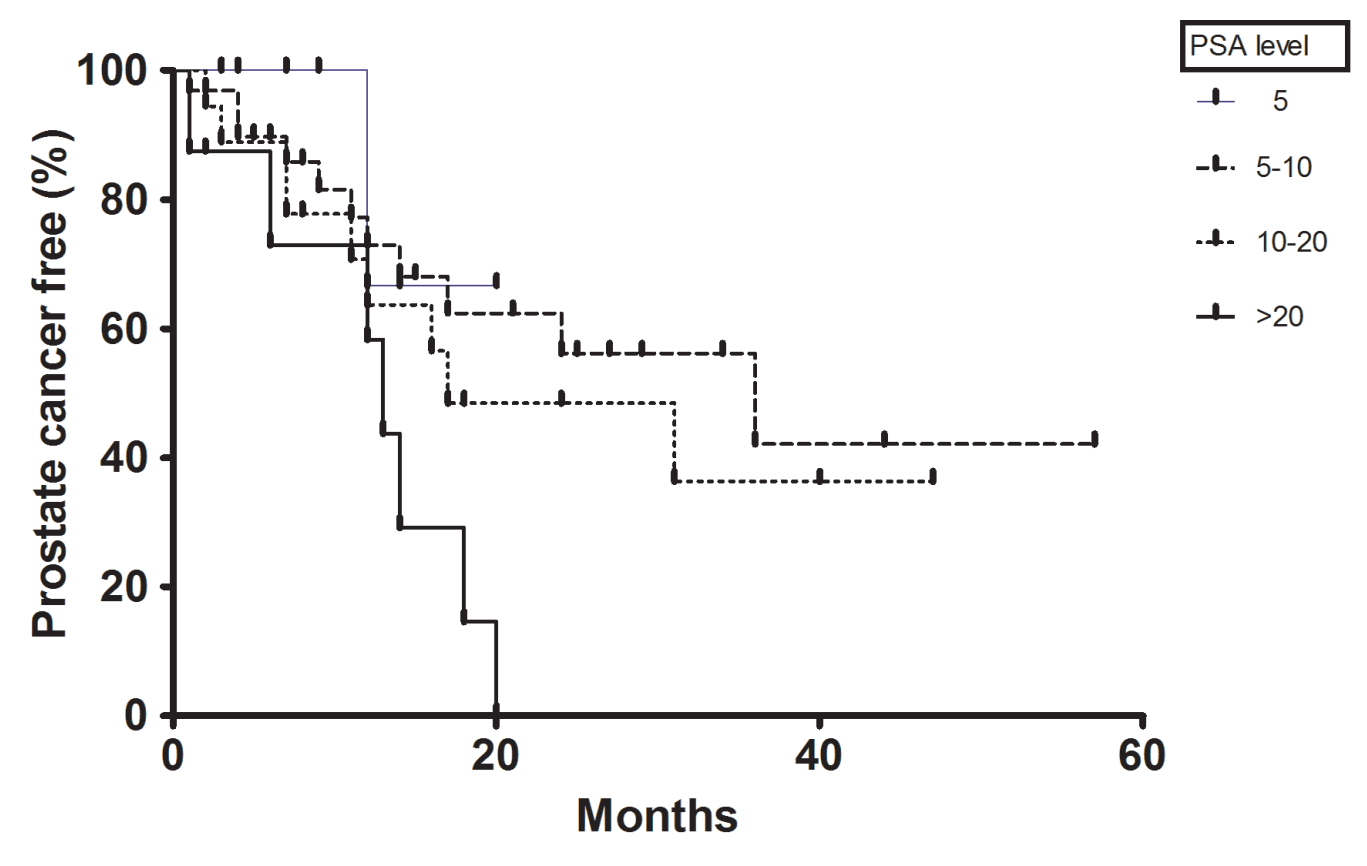
**Prostate cancer-free interval from the time of 1^st ^biopsy, according to PSA levels at presentation**.

We performed a sub-group analysis at different PSA ranges (Table 2; Figure 1). At PSA range ≤ 5 ng/ml there were 15 men with HGPIN; 9 of these underwent repeat biopsies at a mean interval of 6 m (range 1 to 14 months). Two had one further set of biopsies and in 1, CaP was diagnosed. There was virtually no change in the PSA (mean change of 0.3 ng/ml) in these men who had repeat biopsies. For those with presenting PSA between 5 and 10 ng/ml, CaP was diagnosed in 11 men of the 32 who had repeat biopsies. The repeat biopsies were performed at a mean interval of 16.7 months (range 1 to 36 months) while the mean change in PSA was only 1.3 ng/ml in this cohort of men. The cancer detection rate was markedly higher in men with PSA ≥ 10 ng/ml. For PSA range > 10 ≤ 20 ng/ml, it was 50%; in those with presenting PSA > 20 ng/ml, it was 87.5%.

**Table 2:** Detection rates of prostate cancer in men with HGPIN based on the baseline PSA level at first biopsy

**PSA range (ng/ml)**	**Mean PSA in ng/ml (range)**	**No. men with HGPIN**	**Mean age [y (range)]**	**No. undergoing repeat biopsies**	**CaP diagnosis**	**Percentage with CaP diagnosis†**
≤ 5	3.3 (1.5 to 5)	15	61.7 (52 to 74)	9	1	11
> 5 ≤ 10	7.1 (5.2 to 9.5)	37	65.4 (52 to 81)	32	11	34
> 10 ≤ 20	13.5 (10.1 to 19.7)	28	70.1 (61 to 86)	18	9	50
> 20	34.5 (21.3 to 68.2)	8	73.7 (62 to 84)	8	7	87.5

**† **Calculated as a percentage of those that underwent repeat biopsies and were subsequently detected with CaP

## Discussion

Recent reviews on HGPIN have found that it has a median incidence of 5.2% (mean of 7.7%) on needle biopsies. From data derived from recent studies, in men who are identified to have isolated HGPIN on a 1^st ^set of biopsies, there is a median elevated risk of 24% (mean 31.5%) of subsequent CaP on repeat needle biopsies [5,6]. Two-thirds of the 22 contemporary studies (since 2000) reviewed reported the median elevated risk of subsequent CaP to be below 24% [6]. In fact, with the widespread implementation of extended core (≥ 8) biopsies that result in a more rigorous examination of the lateral regions of the prostate, it has been suggested that identification of isolated HGPIN may no longer be associated with higher CaP risk on repeat biopsies. Thus, the CaP-detection rate post-HGPIN on 1^st ^set of biopsies may not be markedly dissimilar to that on repeat biopsies following an original benign diagnosis [5,10].

There appears to be no common consensus regarding the optimal biopsy technique and schedule for repeat biopsies after isolated HGPIN diagnosis on 1^st ^set of biopsies. Three to six monthly repeat biopsies for 2 y followed by yearly biopsies for life has been recommended [11]. Some studies have shown a low detection rate when biopsies are repeated within 12 months after isolated HGPIN on extended core 1^st ^biopsies [6,10]. Lefkowitz *et al*. (2002) looked at follow-up biopsies at 3 years that showed a CaP-detection rate of 25.8%; based on these results they recommended delaying the repeat biopsies for 3 years [12]. There has also been a suggestion for site-directed repeat biopsies after isolated HGPIN on extended core 1^st ^biopsies [13]. Men with multifocal isolated HGPIN have been implicated to be at greater risk of developing CaP in some studies [13,14].

Our current UK-based study showed a 41.8% risk of subsequent diagnosis CaP in men with an original diagnosis of isolated HGPIN on 1^st ^set of biopsies, which is higher than most contemporary studies examining extended core repeat biopsy techniques [6]. However, this rate is comparable to earlier U.S.-based study from the mid-1990s when there was lower level of PSA screening and fewer cores were sampled on prostate biopsies [15].

The sub-analysis based on PSA ranges showed that the elevated risk of CaP detection was only observed at higher PSA ranges. This would suggest two important findings. Firstly, as purely isolated HGPIN should not raise the PSA so patients with low age specific PSA (especially in the PSA range ≤ 5 ng/ml) may be appropriate candidates for repeat biopsies at longer intervals as suggested by Lefkowitz *et al*. (2002) [12]. Secondly, the negative 1^st ^biopsy in the presence of a high PSA may be a reflection of a missed concurrent focus of CaP and in these patients a repeat biopsy strategy at a shorter interval may be more appropriate. A similar strategy in the intermediate PSA range (between 5 to 10 ng/ml) may be justified as the risk of subsequent diagnosis of CaP on repeat biopsies seems to be still high (Table 2; Figure 1). We present part of the data in the Kaplan-Meier type plot (Figure 1); it is important to realise that as the Kaplan-Meier method uses a finite number of observations to provide information for estimation purposes, one should use caution in deducing important conclusions based solely from the curve.

There are a number of limitations to the current study. Either due to patient choice or due to their co-morbidities or for some unknown reasons, 21 patients with diagnosis of HGPIN on first set of biopsies did not undergo repeat biopsies at our institution. Though these patients were relatively older with a mean age of 73 y, no significant difference in the mean PSA level or mean number of biopsy cores at 1^st ^set of biopsies was observed when compared to those that had repeat biopsies (data not shown). In 18 of these 21 patients the clinic follow-up data was available and none of these men have shown rapid rise in PSA or clinical progression to CaP. Also, there does not appear to have been a uniform protocol for repeat biopsies. Furthermore, this is a retrospective study looking at a regional population and, thus may not be representative of the situation in the UK as a whole. Prospective or retrospective studies looking at multiple regions may be needed to examine this more fully.

## Conclusion

In this UK-based study a presentation of isolated HGPIN at 1^st ^set of extended core needle prostate biopsies was associated with a high risk (41.8%) of a subsequent diagnosis of CaP on repeat biopsies. Patient age, higher PSA level at isolated HGPIN diagnosis and higher mean changes in serum PSA levels were predictive of CaP detection on repeat biopsies. Based on the results of our study, we recommend delaying the first repeat biopsy at low PSA range and repeat biopsies at shorter interval at intermediate and higher PSA ranges.

## Competing interests

The authors declare that they have no competing interests.

## Authors' contributions

PBS, NR and FLM conceived the idea of the study. PBS and CMN collected data. PBS drafted the manuscript. PBS and NR did data analysis. CMN, RAB, FLM and SSM helped with manuscript drafting and critical analysis. All authors read and approved the final manuscript.

## Pre-publication history

The pre-publication history for this paper can be accessed here:


